# Physical Activity, Aerobic Capacity, and Total Antioxidant Capacity in Healthy Men and in Men with Coronary Heart Disease

**DOI:** 10.1155/2015/197307

**Published:** 2015-09-14

**Authors:** Anna Gawron-Skarbek, Jacek Chrzczanowicz, Joanna Kostka, Dariusz Nowak, Wojciech Drygas, Anna Jegier, Tomasz Kostka

**Affiliations:** ^1^Department of Geriatrics, Medical University of Lodz, Plac Hallera 1, 90-647 Lodz, Poland; ^2^Department of Preventive Medicine, Medical University of Lodz, Żeligowskiego Street 7/9, 90-752 Lodz, Poland; ^3^Department of Hygiene and Health Promotion, Medical University of Lodz, Jaracza Street 63, 90-251 Lodz, Poland; ^4^Cardiac Rehabilitation Centre, Copernicus Memorial Hospital, Popioły Street 40, 93-438 Lodz, Poland; ^5^Department of Physical Medicine, Medical University of Lodz, Plac Hallera 1, 90-647 Lodz, Poland; ^6^Department of Clinical Physiology, Medical University of Lodz, Mazowiecka Street 6/8, 92-215 Lodz, Poland; ^7^Department of Sports Medicine, Medical University of Lodz, Pomorska Street 251, 92-213 Lodz, Poland

## Abstract

*Objective.* The purpose of the study was to assess total antioxidant capacity (TAC) of blood serum in relation with habitual leisure time physical activity (LTPA) and aerobic capacity in a group of 90 men with coronary heart disease (CHD) aged 34.8–77.0 years and in 90 age-matched peers without CHD.* Methods.* Two spectrophotometric methods were applied to assess TAC: Ferric Reducing Ability of Serum (TAC-FRAS) and 2.2-diphenyl-1-picryl-hydrazyl (TAC-DPPH) tests. Aerobic capacity was expressed as physical working capacity at 85% of the maximal heart rate (PWC_85%HRmax_).* Results*. CHD patients had higher values of TACFRAS (1.37 ± 0.28 versus 1.27 ± 0.23 mmol FeCl_2_·L^−1^; *P* < 0.05) but there were no group differences for TAC-DPPH and for uric acid (UA). Negative correlation was found between LTPA (also when calculated per kg of body mass) and TAC-DPPH in CHD patients. In CHD patients, TAC-FRAS and UA were lower in subjects with higher aerobic capacity expressed as PWC_85%HRmax/kg_. Those associations were not found in healthy men.* Conclusions*. We conclude that TAC of blood serum is moderately adversely related to LTPA and aerobic capacity in patients with CHD. UA, as the main determinant of serum TAC, may be partially responsible for those associations.

## 1. Introduction

Numerous studies focus on the role of antioxidant potential and reactive oxygen species in the aging process as well as in the pathogenesis of common civilization diseases, such as cardiovascular diseases [1, 2]. Under specific physiological conditions associated with an increasing imbalance of free radicals and antioxidants, in favor of the former, a chronic state of oxidative stress may occur [3–5]. Reactive oxygen species are essential to the normal cell functioning, but coexistent antioxidant defenses are necessary to avoid the damaging effects of excessive free radicals production. It has been suggested that higher antioxidant potential can protect the organism against undesirable reactive oxygen species activity and thus prevent disease incidence [1, 2]. However, the results of available data are full of discrepancies and the present state of knowledge on such dependence is still not complete [6, 7]. It is not evidenced whether the incidence of coronary heart disease (CHD) is related to a decrease in antioxidant potential. Relationship of CHD to antioxidant defenses may be modified by many demographic, anthropometric, physiological, and biochemical confounders [8, 9]. Some data indicate that physical activity (PA) and aerobic capacity (fitness) may be important determinants of antioxidant status among both CHD and healthy subjects [10]. Regular PA at moderate intensity may induce favorable changes in antioxidant defense system, especially by an increase of individual enzymatic antioxidants activity [11, 12]. On the other hand, unfavorable effects may occur during too vigorous PA, inducing free radical damages in different tissues [13]. Therefore, antioxidant potential assessment should include characteristics of PA applied together with the characteristics of the subjects like age, health status, and other potentially modifying factors. These results seem to be crucial for planning cardiac rehabilitation in CHD patients.

Total antioxidant capacity (TAC) assessment is an established methodology of simultaneous measurement of different elements of antioxidant defense system. Several methods for TAC measurement are available including Ferric Reducing Ability of Serum (FRAS), being a modification of the ferric reducing ability of plasma (FRAP) [14] and a new spectrophotometric 2.2-diphenyl-1-picryl-hydrazyl (DPPH) test [15].

There is a scarcity of data analyzing the influence of PA and fitness on antioxidant potential in cardiac diseases considering other potential modifiers. Therefore, the aim of the present study was to compare the potential impact of PA and aerobic capacity on TAC in CHD patients and in healthy age-matched subjects, taking into account other cardiovascular risk factors.

## 2. Methods

### 2.1. Subjects

The study was carried out in the two age-matched groups of men. The first studied group (I) comprised CHD patients who were participating in a cardiac rehabilitation program in the Cardiovascular and Metabolic Diseases Prevention Centre and Ambulatory Rehabilitation Centre of the Central Clinical Hospital of the Medical University in Lodz (Poland), whereas the second control group (II) was composed of relatively healthy men, without CHD, registered in the Healthy Man Centre of the Medical University of Lodz.

The group I consisted of 90 CHD patients aged 38.3–77.0 (56.63 ± 7.64) years in whom the most recent acute coronary event, cardiac or cardiosurgery intervention, had occurred a minimum of one month earlier. Among the males with CHD 72 had a history of myocardial infarction (7 patients, twice), 58 men demonstrated arterial hypertension, and 12 had diabetes mellitus. An applied pharmacotherapy regimen usually involved aspirin (*n* = 78), statins (*n* = 78), fibrates (*n* = 2), beta-blockers (*n* = 71), angiotensin-converting enzyme (ACE) inhibitors (*n* = 39), ticlopidine (*n* = 7), long-acting nitrates (*n* = 25), diuretics (*n* = 13), calcium channel blockers (*n* = 10), oral antidiabetic drugs (*n* = 11), and insulin (*n* = 1).

All the CHD patients were qualified for cardiac rehabilitation aerobic type exercise training (3 times a week) and the majority (68 subjects) participated regularly in exercise sessions. Exercise training sessions were run either on a bicycle ergometer (45 minutes) or in the gym (60 minutes: conditioning exercises, brisk walking, and volleyball played with several safety rules, e.g., obligatory 3 hits before returning a ball to another side and no jumps to block a ball). A few individuals were individually taking up some noncompetitive endurance type activities like walking, jogging, cycling, or swimming. Some inactive CHD patients did not participate in the proposed cardiac rehabilitation program despite the lack of medical contraindications.

To every patient, an age-matched peer without CHD was assigned. Group II comprised 90 relatively healthy males aged 38.3–76.1 (56.79 ± 7.45) years. All the men were either sedentary or were involved in recreational noncompetitive endurance sports, for example, running, cycling, swimming, volleyball, and basketball. Fourteen of these men had arterial hypertension and were treated with beta-blockers (*n* = 4) and ACE inhibitors (*n* = 12). Eight men were treated for hypercholesterolemia with statins and 8 used preventive treatment with low-dose aspirin.

All the subjects in the study were free from known malignant diseases, important chronic inflammatory diseases, disability, or dementia. Apart from salt, glucose, and cholesterol limitations, none of the subjects was following a special diet. All the subjects were informed of the purpose of the study. The study had been approved by the Ethics Committee and written informed consent was obtained from all the subjects.

### 2.2. Protocol and Measures

The examinations took place in the Department of Geriatrics and the Department of Preventive Medicine. Laboratory measurements were performed both in the Department of Preventive Medicine and in the Department of Clinical Physiology, Medical University of Lodz [16]. The subjects were asked to report to the centre between 8.00 and 9.00 a.m. after overnight fasting for a minimum of 12 hours (they could consume a light supper without animal-derived fats the previous day, but no other particular dietary instructions were given), after overnight rest, restraining from physical exercises, smoking, and alcohol for at least 12 h before laboratory measurements. After fasting blood drawing all the participants were given a light breakfast and a multidimensional assessment was performed with each subject. During the standard medical interview habitual PA level was assessed and the smoking habit, alcohol consumption, and usual dietary habits were evaluated using the World Health Organization Countrywide Integrated Noncommunicable Diseases Intervention (WHO CINDI) questionnaire [17].

### 2.3. Anthropometric Data

Anthropometric data was collected by standard methods. Height and weight were measured and the body mass index (BMI) (kg·m^−2^) was calculated. Skinfold measurements were taken at four sites: triceps, biceps, subscapula, and supraileum. The percentage of body fat was estimated from skinfold measurements according to Durnin and Womersley [18]. Measurements of waist and hip circumference were taken and waist-to-hip ratio (WHR) was calculated as an index of visceral obesity.

### 2.4. Physical Activity Assessment

Habitual PA level assessment was performed as a standard face to face questionnaire interview. The level of energy expenditure related to habitual leisure time physical activity (LTPA) was estimated. On the basis of the number of hours and minutes earmarked for weekly rehabilitation/recreational/sports activities (the approximate metabolic costs of leisure time sports activities, expressed in kcal·week^−1^) according to the tables of Fox et al. [19], the value of energy expenditure was calculated. The subjects were asked to sum up all the rehabilitation/recreational/sports physical activities for one week before blood sample collection.

### 2.5. Physical Fitness (Aerobic Capacity)

The graded submaximal exercise test was performed after medical qualification that involved physical examination and electrocardiogram at rest (12-lead ECG at rest conducted with Farum Multicard E330 instrument). It was carried out on a Monark type 818E (Stockholm, Sweden) bicycle ergometer with 30-Watt increments every 3 minutes to achieve at least 85% (75% for men taking beta-adrenergic blockers) of maximal age-predicted heart rate (HR) (220, age) or when symptoms imposing the test discontinuation occurred. HR (continuous ECG tracing) was regressed against the three last workloads. The resultant linear regression equation was used to calculate the aerobic capacity index, that is, physical working capacity at 85% of the maximal HR (PWC_85%HRmax_). PWC_85%HRmax_ was calculated by interpolating the workload-HR regression line (with at least 3 workloads) at the point of 85% of the maximal age-predicted HR and at the point of 75% of the maximal age-predicted HR for men taking beta-adrenergic blockers. This methodology, even with lower (PWC_75%HRmax_) exercise test intensity level, has been proposed as a useful measure of aerobic power in epidemiological studies [20]. PWC_85%HRmax_ was expressed as absolute values (PWC (W)) and relative to body mass (PWC per kilogram (W·kg^−1^)).

### 2.6. Laboratory Measurements

Fasting blood samples were drawn from the antecubital vein: for measurements of TAC into Vacuette tubes (Greiner Bio One GmbH, Kremsmunster, Austria) with sodium heparin (200 mg·L^−1^) or into siliconized tubes (for other tests). Enzymatic methods were used to determine serum total cholesterol (TC), triglycerides (TG), glucose, and uric acid concentration (UA) (CORMAY Liquick Cor-CHOL, Liquick Cor-TG, Liquick Cor-GLUCOSE, and Liquick Cor-UA, resp.). High density lipoprotein cholesterol (HDL-C) was measured by the precipitation method (CORMAY-HDL). Low density lipoprotein cholesterol (LDL-C) was estimated using the Friedewald formula [21].

### 2.7. Total Antioxidant Capacity

Blood samples were incubated for 30 minutes at 37°C and then centrifuged for 10 minutes (4°C, 1500 ×g) for further TAC measurements. Subsequently the samples were stored at −80°C for no longer than 30 days prior to the assays of antioxidant activity [15, 22]. Measurements of blood serum TAC were performed using two spectrophotometric methods: the FRAS method (Ferric Reducing Ability of Serum) originally described by Benzie and Strain [14] with some modifications [15] and the DPPH method (2.2-diphenyl-1-picryl-hydrazyl) [15, 22]. To get reliable data, all individual results were calculated as a mean from three separate measurements. Mean coefficients of variation across the triplicate measurements (*n* = 30) calculated for TAC-DPPH and TAC-FRAS were 0.049 and 0.018, respectively. In addition, both TAC assessments were performed in parallel using the same laboratory equipment (spectrophotometer) and within the same time frame. The FRAS test measures TAC determined by nonenzymatic antioxidants; the main contributors are ascorbic acid and UA, whereas plasma proteins (e.g., albumins) and low molecular weight thiols (e.g., reduced glutathione, GSH) have very low activity in this method [14, 23]. TAC-FRAS values are expressed in mmol·L^−1^ of formed FeCl_2_ (mmol FeCl_2_·L^−1^). DPPH test consists of the scavenging of free radical DPPH (a relatively stable compound in alcoholic solution with a peak absorbance at *λ* = 517 nm) by a complex of antioxidants in the assayed sample of deproteinized serum. A decline of absorbance values equivalent to % of DPPH reduction expresses level of TAC-DPPH. The precise methodology of both tests has been described elsewhere [15, 16].

### 2.8. Statistical Analysis

Data were verified for normality of distribution and equality of variances. Variables that did not meet the assumption of normality were analyzed with nonparametric statistics. A one-way analysis of variance (ANOVA) with Bonferroni post hoc testing, the Kruskal-Wallis test, and chi-square test (with Yates' correction for 2 × 2 tables) were used for comparison between the two groups of men. Pearson product moment or Spearman correlations were used to determine the relationships between variables. Those PA and fitness variables statistically significant (*P* value < 0.05) in the bivariate analyses were further analyzed in a multivariate regression (general linear model) adjusted for age and BMI (model I) and for age, BMI, and UA (model II) to verify variables that independently predict TAC level in CHD group. LTPA was transformed to categorical variable (satisfactory versus unsatisfactory LTPA level) in multivariate approach according to the recommendations of the European Association of Cardiovascular Prevention and Rehabilitation [24]. Thirty CHD patients fulfilling minimal (≥1000 kcal/week) LTPA recommendations were classified as high, and 60 patients with low LTPA (<1000 kcal/week) were coded as low LTPA. PWC_85%HRmax/kg_ and TAC-FRAS values were normalized using a log transformation and TAC-DPPH values were normalized using a square root transformation for the purpose of multivariate statistical analyses. The results were presented as the mean ± standard deviation. The level of significance was set at *P* < 0.05 for all analyses.

## 3. Results

Table 1 shows baseline characteristics of both groups: age, selected anthropometric and biochemical characteristics, prevalence of smoking, and blood pressure. Men with CHD were characterized by higher values of body mass, BMI, waist circumference, WHR, and percentage of body fat in comparison with men without CHD. Among the patients with CHD, significantly more subjects had smoked in the past than in the group of men without CHD. Values of both systolic (SBP) and diastolic blood pressure (DBP) did not differ between the groups. CHD patients had lower values of TC, LDL-C, and HDL-C concentrations and lower TC/HDL-C ratio. There were no group differences for TG, glucose, and UA concentrations (Table 1).

Table 2 shows LTPA, fitness measures, and TAC values in both groups. Healthy men were characterized with significantly higher level of energy expenditure corresponding to LTPA and higher values of PWC_85%HRmax_ and PWC_85%HRmax/kg_ than men with CHD. CHD subjects had higher values of TAC-FRAS (1.37 ± 0.28 versus 1.27 ± 0.23 mmol FeCl_2_·L^−1^; *P* < 0.05) but there were no group differences for TAC-DPPH (10.2 ± 3.5 versus 11.3 ± 4.6% reduction) (Table 2).

**Figure 1:**
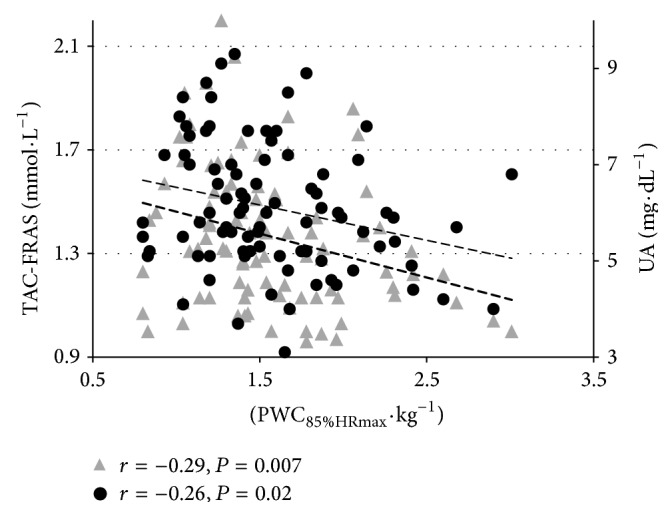
Correlation between TAC-FRAS and PWC_85%HRmax_·kg^−1^ and between UA and PWC_85%HRmax_·kg^−1^ in CHD patients.

Correlations between TAC, UA, and current LTPA and aerobic capacity indices for both groups are presented in Table 3. Negative correlation was found between LTPA (also when calculated per kg of body mass) and TAC-DPPH in CHD patients. Moreover, in CHD patients, TAC-FRAS (*r* = −0.29; *P* = 0.007) and UA (*r* = −0.26; *P* = 0.02) were lower in subjects with higher aerobic capacity expressed as PWC_85%HRmax/kg_ (Figure 1). Those associations were not found in healthy men. Both TAC measures were related to UA in either group (Table 3).

In both groups, PA and fitness measures were generally negatively related to body fatness indicators, TC, LDL-C, TC/HDL ratio, triglycerides, glucose levels, and blood pressure, while being positively related to HDL-C. In CHD group, TAC was directly related to the majority of body fatness measures, with the highest relationship with BMI (*r* = 0.38 for TAC-FRAS and *r* = 0.25 for TAC-DPPH). In healthy men, TAC-FRAS was related to WHR (*r* = 0.22; *P* = 0.04). Age and other biochemical and blood pressure measurements were not related to TAC in either group. TAC-FRAS and TAC-DPPH were also strongly intercorrelated both in CHD group (*r* = 0.33) and in healthy men (*r* = 0.43) (not shown in the table).

### 3.1. Multivariate Analyses

Those PA and fitness variables statistically significant (*P* value < 0.05) in the bivariate analyses were further analyzed in a multivariate regression adjusted for age and BMI (model I) and for age, BMI, and UA (model II) to verify variables that independently predict TAC level in CHD group.

After adjustment for age and BMI, log PWC_85%HRmax/kg_ remained statistically significantly related to log TAC-FRAS (*P* = 0.027). After adjustment for age, BMI, and UA, log PWC_85%HRmax/kg_ was not any longer related to log TAC-FRAS (*P* = 0.26), with UA (*P* < 0.001) being the only independent predictor of log TAC-FRAS (adjusted *R* ^2^ = 41.6).

Thirty CHD patients with satisfactory minimal (≥1000 kcal/week) LTPA had lower TAC-DPPH as compared to 60 patients with low LTPA (2.94 ± 0.51 versus 3.24 ± 0.60; *P* = 0.009). After adjustment for age and BMI in general linear model, the association of squared root TAC-DPPH to LTPA level was still significant (*P* = 0.038). After adjustment for age, BMI, and UA, LTPA was still related to TAC-DPPH (*P* = 0.037), with UA (*P* = 0.003) and LTPA level being the only independent predictors of TAC-DPPH variability (adjusted *R* ^2^ = 15.3).

## 4. Discussion

Available data concerning influence of PA and physical fitness on the level of antioxidant barrier is full of discrepancies. Some reports show a positive effect of systematic PA on antioxidant potential [4, 25–32], others note a lack of this association [33–35], and finally there are studies which demonstrate adverse effect of PA on antioxidant capacities [36–38]. In a cross-sectional population study a negative relationship of physical fitness to TAC was found in females [36]. Also, the results of current work indicate that more active and fit subjects, whilst having more favorable cardiometabolic diseases risk profile, are characterized with lowered antioxidant capacities. Nevertheless, this relation was observed only among CHD men, not among healthy ones. By contrast, a significant positive correlation between plasma antioxidant concentrations and physical performance in the elderly was observed by Cesari et al. [30]. Similarly, higher PA resulted in decreased plasma oxidative stress markers (iso-PGF2*α* and protein carbonyl concentration) and increased erythrocyte superoxide dismutase (SOD) activity in older subjects [39]. Then, in the ATTICA study, TAC was positively correlated with the level of PA [25]. The previous studies of Kostka et al. did not indicate any relations between TAC and PA/fitness status in healthy active elderly women [40] or men [35]. However, the negative correlations for the red blood cell glutathione peroxidase (GSH-Px) with aerobic fitness and PA indices were found in women [40]. Also, years of training in competitive rowers had a small inverse correlation with TAC and a small association with the exercise-induced change in TAC [41].

In the present report, several negative correlations between TAC and LTPA and between TAC and aerobic capacity were observed in CHD group. These associations were further verified after adjustment for potential confounders: BMI as the most powerful index of overweight/obesity increasing TAC and UA as the main determinant of TAC of blood serum [16]. The observed LTPA-TAC associations remained significant after adjustment for age and body composition (BMI) and for physical fitness were blunted after adjustment for UA levels. In healthy men none of those correlations was identified. Nevertheless, in a much larger group of healthy men similar relationships were observable [38]. Single exercise session is related to increased reactive oxygen species production and transient depletion of antioxidant potential [42]. As compared to healthy subjects, CHD patients may be more vulnerable to exercise training, with more blunted recovery process, so exercise overloading leads to redoubled antioxidants consumption and to TAC decrease. The depletion of antioxidant barrier may also be an effect of sustained oxidative stress related to presence of common cardiometabolic risk factors (overweight/obesity, long-lasting smoking habit, or laboratory lipid indices of metabolic syndrome) [38, 43]. On the other hand, in the course of many diseases, in particular in early stages of disorders or during illness intensification or after some medical intervention (e.g., applied pharmacotherapy or the beginning of cardiac rehabilitation), the antioxidant defense system may respond by increasing its activity [42].

Understanding the mechanisms responsible for optimal preventive and therapeutic influence of PA and for reduction of potential negative effects of oxidative stress seems to have crucial meaning for future studies [44]. One of possible explanations for the association between unfavorable overall cardiometabolic risk profile and higher TAC may be based on the classical physiological concept of hormesis. This Greek term means a state in which a sublethal dose of toxin can increase the tolerance of organism to stand up to higher doses of toxin [45]. Regarding this theory, the best strategy to enhance endogenous antioxidants level may actually be the oxidative stress itself but in “appropriate dose” [46]. It means that overweight/mild obesity or sedentary lifestyle may stimulate TAC and high values of antioxidant potential among obese and unfit individuals may be explained by secondary response to intensified oxidative stress characterizing inactive subjects with higher amount of adipose tissue [47, 48]. Therefore, these two phenomena should not be separated but rather seen as a sequence of incidents.

One of those compensatory oxidative stress-connected mechanisms stimulating antioxidant defense system may be related to elevated UA concentration. There is some evidence that increased level of circulating UA may protect against oxidative modification of endothelial enzymes and preserve the ability of the endothelium to mediate vascular dilatation in oxidative stress [49]. Simultaneously, UA possesses another nature as its high concentration is considered as one of the cardiovascular diseases risk factors [50]. Our present data do not show the difference between two groups of men with regard to UA level but higher UA values as well as higher TAC-FRAS values were found in CHD patients who presented lower aerobic capacity level. Associations between TAC and PA/fitness were not visible in individuals without CHD, suggesting that, in CHD patients, the antioxidant defense system is more vulnerable to permanent oxidative stress connected even with relatively low intensity physical efforts during cardiac rehabilitation training sessions. Similarly to our results, in the study of Nishida et al. [51], moderate intensity PA was inversely correlated with UA level in obese individuals. On the other hand, Beavers et al. [52] identified about 5% higher values of UA in older adults after 12-month moderate intensity PA intervention as compared with subjects who participated only in a successful aging health education intervention. Ideally, exercise training should be able to reduce proinflammatory levels of UA to antioxidant and protective levels. Nevertheless, up to now, a wide range of studies was not able to clarify what type, duration, and intensity of exercise training should be taken to maximize the benefits of physical exercise for antioxidant potential in different groups of subjects and patients [53]. In the present study, UA was the strongest predictor of antioxidant status, irrespective of TAC assay and studied group. Similar predominant effect on antioxidant potential has also been noted in other studies [29, 54]. Nevertheless, this two-directional activity of UA, as both potent antioxidant and cardiovascular risk factors, merits further studies.

Several shortcomings of the present study should be acknowledged. This is a cross-sectional case-control study performed in CHD patients and their healthy peers. Our subjects were volunteers, probably more physically active, and more prone to undergo exercise testing than it would be the case in a random sample. Patterns of associations between PA/fitness and TAC may be different in longitudinal comparisons. Although both DPPH and FRAS tests measure the TAC of blood serum, they reflect somewhat different physiological properties [15]. Neither of the methods for TAC assessment measures all the antioxidants occurring in body fluids. That is why using simultaneously the two assays, FRAS and DPPH, in spite of their limitations, enhances a completeness and reliability of measurement. It is generally recommended to use at least two methods of determining TAC due to the differences in the testes used for investigation [22, 23, 55, 56]. The correlation between the used methods (*r* = 0.44; *P* < 0.0001) [15] is higher than among others allowing for the assessment of TAC, for example, FRAP and OXY (*r* = 0.22) [56] and FRAP and ORAC (*r* = 0.35) [23, 55]. Neither of the methods is expensive and they are quick and simple to perform. The values are reproducible and linearly correlated to the concentration of antioxidants present in the samples [15]. Nevertheless, the obtained correlations coefficients of TAC to PA/fitness data were relatively modest. Despite the serious limitations of TAC concept that preclude its meaningful application in vivo, measurement of TAC in biological fluids is regarded as more physiologically representative than the use of individual antioxidants [57]. It is believed to be a useful measure of antioxidant protection against oxidative damage of membranes and other cellular components [58]. On the other hand, limited success of preventive cardiology with the use of general antioxidants will probably stimulate future research towards more site-specific antioxidative therapy [7].

## 5. Conclusions

Our results show that though PA and fitness are related to more favorable cardiometabolic diseases risk profile in men, this impact is not detectable for TAC. In fact, an inverse relationship between PA/fitness and TAC was observed in patients with CHD. UA, as the main determinant of serum TAC, may be partially responsible for those associations.

## Figures and Tables

**Table 1:** Age, selected anthropometric and biochemical characteristics, cigarette smoking, and blood pressure in men with CHD and in their peers without CHD.

Variable	Group I (CHD patients) *n* = 90	Group II (without CHD) *n* = 90
Age (years)	56.63 ± 7.64	56.79 ± 7.45
Body mass (kg)	**85.9 ± 12.3** ^*^	82.5 ± 12.9
BMI (kg·m^−2^)	**28.7 ± 3.7** ^‡^	26.8 ± 3.6
Waist circumference (cm)	**101.4 ± 9.8** ^‡^	94.3 ± 10.0
WHR	**0.97 ± 0.05** ^‡^	0.93 ± 0.06
Percentage of body fat	**25.5 ± 5.2** ^‡^	23.0 ± 5.7
Past smokers (% of *n*)	**78.9** ^‡^	55.6
Current smokers (% of *n*)	15.6	13.3
SBP (mmHg)	126.9 ± 14.1	127.3 ± 13.1
DBP (mmHg)	82.1 ± 8.7	82.2 ± 8.5
TC (mg·dL^−1^)	**187.2 ± 45.2** ^‡^	219.7 ± 46.2
LDL-C (mg·dL^−1^)	**111.0 ± 38.7** ^‡^	145.7 ± 43.0
HDL-C (mg·dL^−1^)	**46.2 ± 10.2** ^*^	49.9 ± 11.4
TC/HDL-C ratio	**4.15 ± 1.26** ^*^	4.62 ± 1.41
TG (mg·dL^−1^)	154.5 ± 108.4	122.2 ± 52.2
Glucose (mg·dL^−1^)	104.7 ± 32.4	98.7 ± 15.3
UA (mg·dL^−1^)	6.11 ± 1.33	6.10 ± 1.22

^*^ *P* < 0.05, ^‡^ *P* < 0.001 as compared to the healthy subjects.

BMI: body mass index, WHR: waist-to-hip ratio, SBP: systolic blood pressure, DBP: diastolic blood pressure, TC: total cholesterol, LDL-C: low density lipoprotein cholesterol, HDL-C: high density lipoprotein cholesterol, TG: triglycerides, UA: uric acid, and CHD: coronary heart disease.

**Table 2:** Physical activity, fitness measures, and TAC in men with CHD and in their peers without CHD.

Variable	Group I (CHD patients) *n* = 90	Group II (without CHD) *n* = 90
Energy expenditure for LTPA (kcal·week^−1^)	**1187 ± 1668** ^†^	2320 ± 2707
Energy expenditure for LTPA (kcal·week^−1^·kg^−1^)	**14.1 ± 19.5** ^†^	30.2 ± 36.0
PWC_85%HRmax_ (W)	**133.5 ± 41.1** ^‡^	157.0 ± 36.6
PWC_85%HRmax_/kg (W·kg^−1^)	**1.56 ± 0.47** ^‡^	1.95 ± 0.53
TAC-FRAS (mmol FeCl_2_·L^−1^)	**1.37 ± 0.28** ^*^	1.27 ± 0.23
TAC-DPPH (% reduction)	10.2 ± 3.5	11.3 ± 4.6

^*^ *P* < 0.05, ^†^ *P* < 0.01, and ^‡^ *P* < 0.001 as compared to the healthy subjects.

LTPA: leisure time physical activity, PWC: physical working capacity, HR: heart rate, TAC: total antioxidant capacity, FRAS: Ferric Reducing Ability of Serum, DPPH: 2.2-diphenyl-1-picryl-hydrazyl, and CHD: coronary heart disease.

**Table 3:** Correlation coefficients of total antioxidant capacity measures and uric acid concentration to physical activity and fitness characteristics in men with and without CHD.

Variable	Group I (CHD patients)			Group II (without CHD)		
	TAC-FRAS (mmol FeCl_2_·L^−1^)	TAC-DPPH (% reduction)	UA (mg·dL^−1^)	TAC-FRAS (mmol FeCl_2_·L^−1^)	TAC-DPPH (% reduction)	UA (mg·dL^−1^)
Energy expenditure for LTPA (kcal·week^−1^)	−0.12	−**0.24** ^*^	−0.04	0.004	−0.03	−0.13
Energy expenditure for LTPA (kcal·week^−1^·kg^−1^)	−0.14	−**0.23** ^*^	−0.04	0.03	0.004	−0.16
PWC_85%HRmax_ (W)	−0.16	−0.0008	−0.14	−0.06	−0.02	−0.01
PWC_85%HRmax/kg_ (W·kg^−1^)	−**0.29** ^†^	−0.07	−**0.26** ^*^	−0.09	0.03	−0.16
UA (mg·dL^−1^)	**0.60** ^†^	**0.37** ^†^	—	**0.64** ^†^	**0.30** ^†^	—

^*^ *P* < 0.05, ^†^ *P* ≤ 0.01.

UA: uric acid, LTPA: leisure time physical activity, PWC: physical working capacity, HR: heart rate, TAC: total antioxidant capacity, FRAS: Ferric Reducing Ability of Serum, DPPH: 2.2-diphenyl-1-picryl-hydrazyl, and CHD: coronary heart disease.

## Abbreviations

PA: Physical activity
BMI: Body mass index
WHR: Waist-to-hip ratio
SBP: Systolic blood pressure
DBP: Diastolic blood pressure
TC: Total cholesterol
LDL-C: Low density lipoprotein cholesterol
HDL-C: High density lipoprotein cholesterol
TG: Triglycerides
UA: Uric acid
LTPA: Leisure time physical activity
PWC: Physical working capacity
HR: Heart rate
TAC: Total antioxidant capacity
FRAS: Ferric Reducing Ability of Serum
DPPH: 2.2-Diphenyl-1-picryl-hydrazyl
CHD: Coronary heart disease.

## References

[1] Maxwell S. R. J. (1995). Prospects for the use of antioxidant therapies. *Drugs*.

[2] Briasoulis A., Tousoulis D., Antoniades C., Stefanadis C. (2009). The oxidative stress menace to coronary vasculature: any place for antioxidants?. *Current Pharmaceutical Design*.

[3] Abdilla N., Tormo M. C., Fabia M. J., Chaves F. J., Saez G., Redon J. (2007). Impact of the components of metabolic syndrome on oxidative stress and enzymatic antioxidant activity in essential hypertension. *Journal of Human Hypertension*.

[4] Fatouros I. G., Jamurtas A. Z., Villiotou V. (2004). Oxidative stress responses in older men during endurance training and detraining. *Medicine and Science in Sports and Exercise*.

[5] Gomes E. C., Silva A. N., Oliveira M. R. D. (2012). Oxidants, antioxidants, and the beneficial roles of exercise-induced production of reactive species. *Oxidative Medicine and Cellular Longevity*.

[6] Münzel T., Gori T., Bruno R. M., Taddei S. (2010). Is oxidative stress a therapeutic target in cardiovascular disease?. *European Heart Journal*.

[7] Otani H. (2013). Site-specific antioxidative therapy for prevention of atherosclerosis and cardiovascular disease. *Oxidative Medicine and Cellular Longevity*.

[8] Hutcheson R., Rocic P. (2012). The metabolic syndrome, oxidative stress, environment, and cardiovascular disease: the great exploration. *Experimental Diabetes Research*.

[9] Duthie G. G., Beattie J. A. G., Arthur J. R., Franklin M., Morrice P. C., James W. P. T. (1994). Blood antioxidants and indices of lipid peroxidation in subjects with angina pectoris. *Nutrition*.

[10] Corbi G., Conti V., Russomanno G. (2012). Is physical activity able to modify oxidative damage in cardiovascular aging?. *Oxidative Medicine and Cellular Longevity*.

[11] Radák Z., Taylor A. W., Ohno H., Goto S. (2001). Adaptation to exercise-induced oxidative stress: from muscle to brain. *Exercise Immunology Review*.

[12] Tong T. K., Lin H., Lippi G., Nie J., Tian Y. (2012). Serum oxidant and antioxidant status in adolescents undergoing professional endurance sports training. *Oxidative Medicine and Cellular Longevity*.

[13] Sen C. K. (1995). Oxidants and antioxidants in exercise. *Journal of Applied Physiology*.

[14] Benzie I. F. F., Strain J. J. (1996). The ferric reducing ability of plasma (FRAP) as a measure of ‘antioxidant power’: The FRAP assay. *Analytical Biochemistry*.

[15] Chrzczanowicz J., Gawron A., Zwolinska A. (2008). Simple method for determining human serum 2,2-diphenyl-1-picryl-hydrazyl (DPPH) radical scavenging activity—possible application in clinical studies on dietary antioxidants. *Clinical Chemistry and Laboratory Medicine*.

[16] Gawron-Skarbek A., Chrzczanowicz J., Kostka J. (2014). Cardiovascular risk factors and total serum antioxidant capacity in healthy men and in men with coronary heart disease. *BioMed Research International*.

[17] Stelmach W., Kaczmarczyk-Chałas K., Bielecki W., Drygas W. (2004). The impact of income, education and health on lifestyle in a large urban population of Poland (CINDI Programme). *International Journal of Occupational Medicine and Environmental Health*.

[18] Durnin J. V. G. A., Womersley J. (1974). Body fat assessed from total body density and its estimation from skinfold thickness: measurements on 481 men and women aged from 16 to 72 years. *British Journal of Nutrition*.

[19] Fox S., Noughton J., Corman P. (1972). Physical activity and cardivascular health. III. The exercise prescripsion: frequency and type of activity. *Modern Concepts of Cardiovascular Diseases*.

[20] Gore C. J., Booth M. L., Bauman A., Owen N. (1999). Utility of pwc75% as an estimate of aerobic power in epidemiological and population-based studies. *Medicine & Science in Sports & Exercise*.

[21] Friedewald W. T., Levy R. I., Fredrickson D. S. (1972). Estimation of the concentration of low-density lipoprotein cholesterol in plasma, without use of the preparative ultracentrifuge.. *Clinical Chemistry*.

[22] Schlesier K., Harwat M., Böhm V., Bitsch R. (2002). Assessment of antioxidant activity by using different in vitro methods. *Free Radical Research*.

[23] Cao G., Prior R. L. (1998). Comparison of different analytical methods for assessing total antioxidant capacity of human serum. *Clinical Chemistry*.

[24] Piepoli M. F., Corrà U., Benzer W. (2010). Secondary prevention through cardiac rehabilitation: from knowledge to implementation. A position paper from the cardiac rehabilitation section of the European association of cardiovascular prevention and rehabilitation. *European Journal of Cardiovascular Prevention and Rehabilitation*.

[25] Kavouras S. A., Panagiotakos D. B., Pitsavos C. (2011). Physical activity and adherence to mediterranean diet increase total antioxidant capacity: the ATTICA study. *Cardiology Research and Practice*.

[26] Franzoni F., Ghiadoni L., Galetta F. (2005). Physical activity, plasma antioxidant capacity, and endothelium-dependent vasodilation in young and older men. *American Journal of Hypertension*.

[27] Child R. B., Wilkinson D. M., Fallowfield J. O. L., Donnelly A. E. (1998). Elevated serum antioxidant capacity and plasma malondialdehyde concentration in response to a simulated half-marathon run. *Medicine and Science in Sports and Exercise*.

[28] Dudek I., Kowalczyk P., Fijalkowski P., Kedziora J. (1994). Effect of a submaximal physical exercise on antioxidant enzymes in the erythrocytes of healthy men. *Biology Sports*.

[29] Child R. B., Wilkinson D. M., Fallowfield J. L. (1999). Resting serum antioxidant status is positively correlated with peak oxygen uptake in endurance trained runners. *Journal of Sports Medicine and Physical Fitness*.

[30] Cesari M., Pahor M., Bartali B. (2004). Antioxidants and physical performance in elderly persons: the Invecchiare in Chianti (InCHIANTI) study. *American Journal of Clinical Nutrition*.

[31] Robertson J. D., Maughan R. J., Duthie G. G., Morrice P. C. (1991). Increased blood antioxidant systems of runners in response to training load. *Clinical Science*.

[32] Takahashi M., Miyashita M., Park J.-H. (2013). The association between physical activity and sex-specific oxidative stress in older adults. *Journal of Sports Science and Medicine*.

[33] Smith D. T., Carr L. J., Dorozynski C., Gomashe C. (2009). Internet-delivered lifestyle physical activity intervention: limited inflammation and antioxidant capacity efficacy in overweight adults. *Journal of Applied Physiology*.

[34] García-López D., Häkkinen K., Cuevas M. J. (2007). Effects of strength and endurance training on antioxidant enzyme gene expression and activity in middle-aged men. *Scandinavian Journal of Medicine and Science in Sports*.

[35] Kostka T., Drai J., Berthouze S. E., Lacour J. R., Bonnefoy M. (2000). Physical activity, aerobic capacity and selected markers of oxidative stress and the anti-oxidant defence system in healthy active elderly men. *Clinical Physiology*.

[36] Sharpe P. C., Duly E. B., MacAuley D. (1996). Total radical trapping antioxidant potential (TRAP) and exercise. *Quarterly Journal of Medicine*.

[37] Sharman J. E., Geraghty D. P., Shing C. M., Fraser D. I., Coombes J. S. (2004). Endurance exercise, plasma oxidation and cardiovascular risk. *Acta Cardiologica*.

[38] Chrzczanowicz J., Gawron-Skarbek A., Kostka J. (2012). Physical activity and total antioxidant capacity across an adult lifespan of men. *Medicine and Science in Sports and Exercise*.

[39] Rowiński R., Kozakiewicz M., Kędziora-Kornatowska K., Hübner-Woźniak E., Kędziora J. (2013). Markers of oxidative stress and erythrocyte antioxidant enzyme activity in older men and women with differing physical activity. *Experimental Gerontology*.

[40] Kostka T., Drai J., Berthouze S. E., Lacour J.-R., Bonnefoy M. (1998). Physical activity, fitness and integrated antioxidant system in healthy active elderly women. *International Journal of Sports Medicine*.

[41] Braakhuis A. J., Hopkins W. G., Lowe T. E. (2013). Effect of dietary antioxidants, training, and performance correlates on antioxidant status in competitive rowers. *International Journal of Sports Physiology and Performance*.

[42] Fisher-Wellman K., Bell H. K., Bloomer R. J. (2009). Oxidative stress and antioxidant defense mechanisms linked to exercise during cardiopulmonary and metabolic disorders. *Oxidative Medicine and Cellular Longevity*.

[43] Opara E. C., Abdel-Rahman E., Soliman S. (1999). Depletion of total antioxidant capacity in type 2 diabetes. *Metabolism: Clinical and Experimental*.

[44] Hamilton K. L. (2007). Antioxidants and cardioprotection. *Medicine and Science in Sports and Exercise*.

[45] Alessio H. M., Hagerman E. A. (2006). *Oxidative Stress, Exercise and Aging*.

[46] Finkel T., Holbrook N. J. (2000). Oxidants, oxidative stress and the biology of ageing. *Nature*.

[47] Keaney J. F., Larson M. G., Vasan R. S. (2003). Obesity and systemic oxidative stress: clinical correlates of oxidative stress in the Framingham Study. *Arteriosclerosis, Thrombosis, and Vascular Biology*.

[48] Morrow J. D. (2003). Is oxidant stress a connection between obesity and atherosclerosis?. *Arteriosclerosis, Thrombosis, and Vascular Biology*.

[49] Becker B. F. (1993). Towards the physiological function of uric acid. *Free Radical Biology and Medicine*.

[50] Suarna C., Dean R. T., May J., Stocker R. (1995). Human atherosclerotic plaque contains both oxidized lipids and relatively large amounts of *α*-tocopherol and ascorbate. *Arteriosclerosis, Thrombosis, and Vascular Biology*.

[51] Nishida Y., Iyadomi M., Higaki Y., Tanaka H., Hara M., Tanaka K. (2011). Influence of physical activity intensity and aerobic fitness on the anthropometric index and serum uric acid concentration in people with obesity. *Internal Medicine*.

[52] Beavers K. M., Hsu F.-C., Serra M. C., Yank V., Pahor M., Nicklas B. J. (2014). The effects of a long-term physical activity intervention on serum uric acid in older adults at risk for physical disability. *Journal of Aging and Physical Activity*.

[53] de Lemos E. T., Oliveira J., Pinheiro J. P., Reis F. (2012). Regular physical exercise as a strategy to improve antioxidant and anti-inflammatory status: benefits in type 2 diabetes mellitus. *Oxidative Medicine and Cellular Longevity*.

[54] Rosell M., Regnström J., Kallner A., Hellénius M.-L. (1999). Serum urate determines antioxidant capacity in middle-aged men—a controlled, randomized diet and exercise intervention study. *Journal of Internal Medicine*.

[55] Prior R. L., Cao G. (1999). In vivo total antioxidant capacity: comparison of different analytical methods. *Free Radical Biology and Medicine*.

[56] Vassalle C., Masini S., Carpeggiani C., L'Abbate A., Boni C., Zucchelli G. C. (2004). In vivo total antioxidant capacity: comparison of two different analytical methods. *Clinical Chemistry and Laboratory Medicine*.

[57] Sies H. (2007). Total antioxidant capacity: appraisal of a concept. *The Journal of Nutrition*.

[58] Ryan M., Grayson L., Clarke D. J. (1997). The total antioxidant capacity of human serum measured using enhanced chemiluminescence is almost completely accounted for by urate. *Annals of Clinical Biochemistry*.

